# Assessing the impact of the COVID‑19 pandemic health protocols on the hygiene status of swimming pools of hotel units

**DOI:** 10.3892/mi.2023.92

**Published:** 2023-06-22

**Authors:** Antonios A. Papadakis, Ioannis Tsirigotakis, Sofia Katranitsa, Christos Donousis, Petros Papalexis, Dimitrios Keramydas, Elias Chaidoutis, Vasiliki Epameinondas Georgakopoulou, Demetrios A. Spandidos, Theodoros C. Constantinidis

**Affiliations:** 1 Laboratory of Hygiene and Environmental Protection, Faculty of Medicine, Democritus University of Thrace, 68100 Alexandroupolis, Greece; 2 Department of Clinical Microbiology and Microbial Pathogenesis, School of Medicine, University of Crete, 71110 Heraklion, Greece; 3 Institute of Agri-Food and Life Sciences, University Research Centre, Hellenic Mediterranean University, 71410 Heraklion, Greece; 4 VELTIA Labs Crete - Be Safer, 71304 Heraklion, Greece; 5 Unit of Endocrinology, First Department of Internal Medicine, Laiko General Hospital, Medical School, National and Kapodistrian University of Athens, 11527 Athens, Greece; 6 Department of Biomedical Sciences, University of West Attica, 12243 Athens, Greece; 7 First Department of Pathology, School of Medicine, National and Kapodistrian University of Athens, Greece; 8 Department of Infectious Diseases and COVID-19 Unit, Laiko General Hospital, Medical School, National and Kapodistrian University of Athens, 11527 Athens, Greece; 9 Laboratory of Clinical Virology, School of Medicine, University of Crete, 71003, Heraklion, Greece

**Keywords:** severe acute respiratory syndrome coronavirus 2, COVID-19, swimming pools, recreational waters, coliforms, *Pseudomonas aeruginosa*, *Legionella spp.*, hygiene, colonization

## Abstract

With the onset of the coronavirus disease 2019 (COVID-19) pandemic, numerous countries imposed strict lockdown measures and travel bans, resulting in the closure of hotels. Over time, the opening of hotel units was gradually allowed, and new strict regulations and protocols were issued to ensure the hygiene and safety of swimming pools in the era of COVID-19. The present study aimed to evaluate the implementation of strict health COVID-19-related protocols in hotel units during the 2020 summer tourist season concerning microbiological hygiene and physicochemical parameters of water, and to compare the data with those from the 2019 tourist season. For this reason, 591 water samples from 62 swimming pools were analyzed, of which 381 samples were for the 2019 tourist season and 210 samples were for the 2020 tourist season. To examine the presence of *Legionella spp*, 132 additional samples were taken from 14 pools, of which 49 in 2019 and 83 in 2020. In 2019, 2.89% (11/381) of the samples were out of legislative limits (0/250 mg/l) regarding the presence of *Escherichia coli* (*E. coli*), 9.45% (36/381) were out of acceptable limits (0/250 mg/l) regarding the presence of *Pseudomonas aeruginosa* (*P. aeruginosa*) and 8.92% (34/381) had residual chlorine levels <0.4 mg/l. In 2020, 1.43% (3/210) of the samples were out of the legislative limits as regards the presence of *E. coli*, 7.14% (15/210) were out of acceptable limits regarding the presence of *P. aeruginosa* and 3.33% (7/210) of the samples measured residual chlorine levels <0.4 mg/l. The risk ratio (RR) in relation to the presence of *E. coli* due to incorrect compliance with the requirements for residual chlorine was calculated for 2019 at 8.50, while in 2020 it was calculated at 14.50 (P=0.008). The RR of the presence of *P. aeruginosa* due to inappropriate residual chlorine requirements was calculated in 2019 at 2.04 (P=0.0814), while in 2020 it was calculated at 2.07 (P=0.44). According to the microbiological hygiene and physicochemical parameters of the water samples studied, there was a significant improvement due to the strict protocols for the swimming pools in the summer season of 2020 compared to the tourist season of 2019, namely 72.72% (*E. coli*), 58.33% (*P. aeruginosa*), 79.41% (of residual chlorine <0.4 mg/l) in the three main parameters studied. Finally, an increased colonization by *Legionella spp.* detected in the internal networks of the hotels due to the non-operation of the hotels during the lockdown, the improper disinfection and stagnant water in the internal water supply networks. Specifically, in 2019, 95.92% (47/49) of the samples tested negative and 4.08% (2/49) tested positive (≥50 CFU/l) for *Legionella spp.*, compared to 2020 where 91.57% (76/83) of the samples tested negative and 8.43% (7/83) tested positive.

## Introduction

In December 2019, China identified the first patient with atypical pneumonia, later known as coronavirus disease 2019 (COVID-19) by the World Health Organization (WHO). A month later, in January 2020, severe acute respiratory syndrome coronavirus 2 (SARS-COV-2), was decoded, identified, and the genome was rapidly published. In mid-April 2020, the effects of COVID-19 on the global population were increasingly evident. The region with the greatest impact per capita was Western Europe, where the elderly, and particularly those with extensive comorbidities and weakened immune systems, were the most affected. Consequently, a more focused approach should be pursued to reduce the impact of COVID-19 in the near term, particularly in the most vulnerable populations, and a longer-term approach should be pursued to reduce the impact of COVID-19 (or other diseases caused by coronavirus) on all populations (1).

SARS-CoV-2 is an enveloped virus with a single-stranded RNA genome that is a member of the coronavirus family. It is comparable to other members of this family, particularly SARS-CoV (also known as SARS-CoV-1), in terms of structure and functionality. Therefore, depending on prior knowledge, the structure, mode of infection, replicative cycle, and type of triggered immune response can be predicted (2). Traditional, intricate and time-consuming methods of vaccine development and production would not have been able to ensure the proper management of the COVID-19 pandemic. As a result, several technologies, including mRNA-based vaccine platforms, have been created for producing vaccines in large quantities and obtaining them rapidly. Although it has made use of prior knowledge and technology, the use of mRNAs in the development of vaccines is not a novel hypothesis (2).

According to the US Centers for Disease Control and Prevention (CDC), there is no evidence that the SARS-CoV-2 virus that causes COVID-19 can spread to humans through recreational waters, such as swimming pools, hot tubs, or fresh or sea water (such as lakes, rivers and oceans). The proper disinfection of water effectively inactivates the virus in water recreation areas. SARS-CoV-2 is transmitted mainly through respiratory droplets and contact with contaminated surfaces; there is no evidence of fecal-oral transmission, which is the main route of infection for most pathogens in recreational waters (3-5). In addition, SARS-CoV-2, as a medium-sized enveloped virus, is more sensitive and is inactivated significantly more rapidly by chlorination than human intestinal viruses without envelope and with known waterborne transmission, such as adenoviruses, noroviruses, rotaviruses and the hepatitis A virus (6).

At the beginning of 2020, a number of countries, including Greece, imposed strict lockdown measures that resulted in the closing of tourist units and swimming pools. At the beginning of June, 2020, hotels in Greece reopened; however, strict restrictive measures were imposed in accordance with laws and regulations issued by the Ministries of Health and Tourism. The use of indoor swimming pools was prohibited in hotels and sports venues, as there were no data as regards their safe use (7,8).

The legislation on the operation of swimming pools in relation to water quality remains outdated, specifically since 1993, with a number of subsequent amendments that did not affect the requirements for the quality characteristics and microbiological hygiene of the water. More specifically, as regards the physicochemical parameters in the water of recreational water installations, the alkalinity of the water is required to be at least 50 mg/l, while the pH values should be maintained between 7.20 and 8.20. The residual chlorine in the tank water measured by the orthotolidine method is required to be at least 0.4 mg/l and preferably not >0.7 mg/l. These values are required to be examined at least twice a day (morning and afternoon) and the results to be recorded in a special book. The water recirculation system of the swimming pools is required to be constantly renewed throughout their operation, at a rate that ensures its complete renewal in not >4 h and, in special cases, 6 h. Renewal must be achieved either by a continuous flow of new, clean water or by recirculating the water in the tanks after prior cleaning and disinfection. Its recirculation-cleaning-disinfection system for water will operate at all times of use of the tanks and beyond for as long as is required to ensure the water is clear and suitable from a microbiological point of view (9-12).

The microbiological quality of the water at the time of operation of the swimming pool should meet the following conditions: i) The number of developing colonies of microbes (in agar after 24 h at 37˚C) should not exceed 200 per ml of water; ii) the number of coliform bacteria should not be >15 per 100 ml water; iii) there are no coliform bacteria [*Escherichia coli* (*E. coli*)] in 100 ml water (13).

During the COVID-19 pandemic, the strict observance of all hygiene and safety rules in the areas of the swimming pools, as well as the strict assurance of water quality, was a key prerequisite for their safe reopening. For this reason, following the issuance of relevant legislative provisions, the requirements were reformulated and specifically: The required sampling for the microbiological quality of the water doubled to at least two samples per week for the period of the first 4 months, a requirement that remained, however, in the next tourist season of 2021. Regular measurement and maintenance of pH records is required every 8 h during the operation of the swimming pools and at least every 2 h during the operation of the hydromassage and hydrotherapy tanks if there is no automatic recording system. The managing bodies of the swimming pools were requested to manually examine chlorine levels during their operation every 4 h for swimming pools and every hour for the water massage tanks and keep a log, unless there is an automatic halogen analyzer and a monitoring system accompanied by an alert system when the parameter values are out of bounds (14,15).

The disinfection of the water and the implementation of strict health protocols remained mandatory in all cases of swimming pool operation, and their proper application is achieved by measuring the residual chlorine in the water of the swimming pools, which will be carried out chromatographically using the N, N-diethyl-p-phenylenediamine method, and the values had to be strictly between 0.4 and 0.7 mg/l. For protection against SARS-CoV-2, it was recommended, according to the instructions of the WHO (https://apps.who.int/iris/handle/10665/43336), that the value of residual chlorine in the tank water be 1-3 mg/l for swimming pools and up to 5 mg/l for water massage tanks.

A subsequent circular of the Ministry of Health in Greece, taking these guidelines into account, recommended that, for precautionary reasons, the value of residual chlorine eventually reach 1.5 mg/l. The frequency of checking for residual chlorine is recommended to be performed four times a day, and the results are to be recorded in a special book. In addition, it was required to observe meticulous cleaning of the swimming pools and their areas (changing rooms, toilets, etc.) with the appropriate cleaning and disinfectant solutions such as sodium hypochlorite 0.1% (1,000 ppm), alcoholic solutions of 70% in ethanol or isopropanol, hydrogen peroxide 0.5%, etc. (8-10).

The CDC reports that in swimming pools, the pH should be maintained between 7.2 and 7.8. They recommend that the concentration of free chlorine should be at least 1 mg/l in swimming pools (ideally 2-4 mg/l). Examining the concentration of free chlorine should be carried out on a regular basis, as free chlorine can be bound by sunlight or depleted in the process of breaking down urine, feces and sweat from the bodies of swimmers. Examining the concentration of free chlorine and pH levels should be carried out at least twice a day and more frequently if the tank is used by more than a few bathers. The majority of bacteria, particularly the bacterium *E. coli*, are killed by free chlorine in <1 min. The hepatitis A virus is neutralized in 16 min, and it can take 45 min to 11 days for some parasites to be killed. According to the CDC, effective preventive measures include the following: i) Regular cleaning and disinfection; ii) frequently touched surfaces should be cleaned and disinfected at least daily; iii) daily testing of pool water for pH and free chlorine levels; iv) maintaining a regular cleaning regime; v) following public hygiene guidelines, such as showering before entering the pool, etc. (16).

Pools and spas are frequently colonized by *Pseudomonas aeruginosa* (*P. aeruginosa*). Furthermore, it is difficult to control the growth of this bacterium due to its ability to form biofilms and interact with other microorganisms. The global risk analysis of pool water is fundamental to identifying appropriate control strategies to prevent or reduce *P. aeruginosa* contamination. Microbiological tests, cleaning surfaces and materials where microorganisms can grow and persist, the showering of users, and controlling the number of pool users are all recommended as essential (17).

Each year, several instances of travel-associated Legionnaires' disease are reported in Europe. Infections can also be linked to hotel activities, such as recreational water activities (swimming pools, spa etc.), in addition to the most obvious locations (cooling towers and hot water systems) (18).

Therefore, the colonization of hotel leisure networks by *Legionella spp.* posed an additional significant risk to bathers' health, particularly during the COVID-19 pandemic era.

The aim of the present study was to evaluate the implementation of strict health COVID-19 protocols in the hotel units of the region of Crete in Greece during the summer tourist season of 2020 concerning microbiological hygiene and physicochemical parameters of water and to compare it with the 2019 tourist season.

## Materials and methods

### Inspections: Sample collection

The material consisted of the 62 swimming pools within hotel units in the region of Crete, from which a total of 591 water samples were collected and analyzed. The 381 samples were collected and analyzed in the tourist season of 2019 and the 210 water samples in the tourist season of 2020, in the context of the self-control of the hotel units. All samples were collected by the 4VELTIA Labs Crete-Be Safer staff. The company maintains a database with all the results per year of the microbiological and chemical analyses, as well as the measurements of the physicochemical parameters of the water for each hotel, in compliance with data protection regulations, which allowed the authors to conduct the present study by comparing data before and after the onset of the COVID-19 pandemic.

The lower number of samples in 2020 can be explained by the fact that the tourist season was delayed due to the COVID-19 pandemic. The samples were collected in accordance with ISO 19458:2006 (https://www.iso.org/obp/ui/#iso:std:iso:19458:ed-1:v1:en).

As regards *Legionella spp.*, the material consisted of the 132 samples collected plus from the 591 samples from 14 swimming pools, of which 49 samples were obtained in 2019 and 83 in 2020. Specifically, the sampling of recreational water was performed using the following methodology: Two samples were obtained from the swimming pools and placed in bottles of 500 ml each. The samples were obtained from the pool's basin at points near outlets (skimmers or gutters). Sodium thiosulfate (Na_2_S_2_O_3_) was used in an 80 mg/l water solution. The sampling points selected were the point of entry into the tank, the point of exit of water, the middle of the tank (20 cm below the water's surface), and before and after the filters. Finally, the samples were labeled and temporarily stored in a cold box at a temperature of up to 5(±3)˚C, protected from direct light, before being delivered to the laboratory immediately after sampling (not >24 h).

### Detection of coliform bacteria and E. coli, Legionella spp. and P. aeruginosa

The enumeration of *E. coli* and coliform bacteria performed according to ISO 9308-1:2014 AMD 1:2016. The method was based on membrane filtration, then cultivation in a chromogenic medium and finally the calculation of the number of target microorganisms in the water sample. This method was particularly suitable for waters with low bacterial counts, which will form <80 colonies on the CCA chromogenic substrate. Such samples were drinking water, disinfected water from swimming pools or treated water. Some strains of *E. coli* that are β-D-glucuronidase-negative, such as *E. coli O157*, will not be detected as *E. coli*. However, if they are β-D-galactosidase-positive, they will appear as coliforms on this chromogenic agar. Coliforms are members of the Enterobacteriaceae family, which express β-D-galactosidase. *E. coli* is a member of the Enterobacteriaceae family, which express β-D-galactosidase, as well as β-D-glucuronidase. The method was based on filtering a specific volume of water sample (usually 100 ml) through a sterile membrane filter with a pore size (0.45 µm) capable of retaining bacteria. The filter was placed on a solid chromogenic substrate (Coliform Chromocult Agar, Chromocult Coliform Agar, 1.10426, Merck Millipore) which is incubated at 36±2˚C for 21 to 24 h.

β-D-galactosidase-positive colonies (pink-red colonies) were then counted as possible *non-E. coli* coliforms. To avoid false-positive results, suspect colonies were confirmed by evidence of a negative oxidase reaction. In addition, β-D-galactosidase- and β-D-glucuronidase-positive colonies (dark blue-violet) were counted as *E. coli*. Total coliforms were the sum of oxidase negative pink-red colonies and dark blue-violet colonies.

*Legionella* was isolated by culture in accordance with the international standard methods (https://www.iso.org/obp/ui/#iso:std:iso:11731:ed-2:v1:en).

Water samples were concentrated by filtration and were re-suspended in distilled deionized water. A volume of the suspension (200 µl) was spread on buffered charcoal yeast extract (BCYE), BCYE minus cysteine (bioMérieux) and glycine vancomycin polymyxin cycloheximide (GVPC) (bioMérieux) Petri dishes: i) Directly after filtration; ii) following incubation at 50˚C for 30 min; iii) following the addition of an acid buffer (0.2 mol/l solution of HCl, pH 2.2 for at least 15 min). The detection limit of the procedure was 50 CFU/l. The inoculated plates were incubated for 10 days at 36±1˚C in 2.5% CO_2_ with increased humidity. Suspected colonies were randomly selected for subculture on BCYE minus cysteine, BCYE and GVPC agar.

For the detection and enumeration of *P. aeruginosa*, the laboratory used the methodology of membrane filtration according to ISO 16266:2006. A measured volume of the water sample (100 ml regularly) or a dilution of the sample, was filtered through a sterile membrane filter of 0.45 µm. The membrane filter was placed on the selective medium and incubated under the conditions specified for the medium (36±2˚C for 44±4 h).

The numbers of presumptive *P. aeruginosa* was obtained by counting the number of characteristic colonies on the membrane filter following incubation. Pyocyanin-producing colonies were considered as confirmed pseudomonas aeruginosa but other fluorescing or reddish-brown colonies require confirmation. Subcultures of colonies requiring confirmation are made from the membrane filter onto plates of nutrient agar. Following incubation at 36±2˚C for 22±2 h, cultures that were not initially fluorescent were tested the oxidase reaction and oxidase positive cultures were tested to produce fluorescein and the ability to produce ammonia from acetamide. Cultures that were fluorescent initially were tested for the ability to produce ammonia from acetamide. From the number of characteristic colonies counted on the membranes and taking account of the proportion of confirmatory tests performed, we calculated the colonies of confirmed *P. aeruginosa* present in the specific volume of water filtered.

### Data collection

Data such as water temperature, pH, residual chlorine concentration, turbidity and alkalinity were recorded during sampling. The inspections were carried out using a checklist developed, collecting information such as name, building address, type of hot water production system, water disinfection system, periodicity and type of maintenance and cleaning of the water supply system, and the number of rooms and beds.

Information such as the date and time of the sampling, the purpose of the sampling, and the name and capacity of the samplers were recorded on the sampling forms. Descriptive data, such as the location of the building, weather conditions, the exact point of sampling, the parameters to be analyzed, the disinfectants, the conditions for sending the sample to the laboratory (under refrigeration, etc.) were also collected.

Measurements were also recorded using calibrated instruments such as temperature, residual chlorine, turbidity, alkalinity and pH. Specifically, the temperatures were measured using a calibrated thermometer placed in the middle of the swimming pool. Free chlorine and pH were measured using a calibrated portable photometer based on a microprocessor. The samples were collected in sterile 1 liter containers containing a sufficient amount of Na_2_S_2_O_3_ (20 mg) to neutralize any chlorine or other oxidizing biocides.

### Statistical analysis

All statistical analyses were conducted using the IBM SPSS Statistics Version 24 statistical package, Epi-Info 2000 version 7.2.5.0 (Centers for Disease Control and Prevention, Atlanta, GA, USA), and the free electronic version of the MedCalc Relative Risk Statistical Calculation Software (19) The relative risk (RR) was calculated with a 95% confidence interval (CI). Analyses to assess categorical risk variables were calculated from water distribution systems and hotel characteristics related to the positive test results. A value of P<0.05 was considered to indicate a statistically significant difference. Pearson's coefficient (r) was used to calculate the linear correlation.

## Results

### Physicochemical parameters of water samples

For the study of the physicochemical parameters of water, the following were considered parametric values: According to the current national legislation, free residual chlorine should be 0.4-0.7 mg/l, while in strict health protocols, it should be 1-1.5 mg/l. The pH values were set between 7.2 and 7.8, turbidity <0.5 NTU, and final alkalinity to 80-120 mg/l (20).

*Residual chlorine levels.* The main parameter measured and whose threshold was legally raised during the pandemic in accordance with Greek legislation and WHO and CDC recommendations was residual chlorine. A total of 591 water samples were measured over both time periods, and these measurements found residual chlorine values of maximum, 8.8 mg/l; minimum, 0.02 mg/l; mean, 1.86±1.84 mg/l; and median, 1.41 mg/l (6,16,21). Of the 591 water samples, 515 (87.14%) were within the limits of national legislation, while 76 (12.86%) were not. In relation to the WHO recommendations, 465 (78.68%) were within limits, while 126 (21.32%) were not.

When comparing the two tourist seasons, in 2019, 321 (84.25%) samples were found within and 60 (15.75%) out of the limits, as determined by the national legislation. In addition, 280 (73.49%) were within and 101 (26.51%) were out of the limits, as determined according to the WHO recommendations.

**Figure 1:**
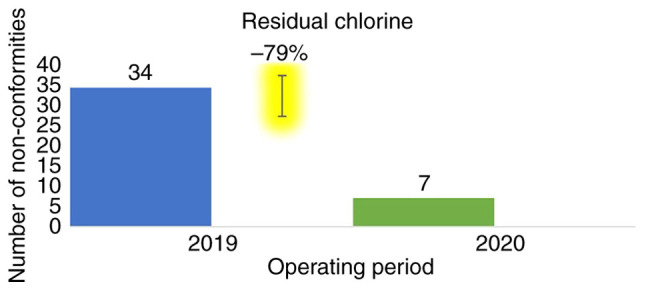
Reduction in non-conformities comparing the years 2019 and 2020 as regards the required residual chlorine.

According to Hellenic legislation (compliance with the guidelines and recommendations of the WHO), in the 2020 tourist season, it was observed that 194 (92.38%) samples were within and 16 (7.62%) out of the limits, and 185 (88.10%) within and 25 (11.90%) out of the limits, respectively,. Compared to the previous year, it appears that there was a 79% reduction in non-conformities regarding the residual chlorine in swimming pools (Fig. 1).

The non-conformities in residual chlorine in the years 2019-2020 for the parametric values of 0.4, 0.7, 1 and 1.5 mg/l are presented in Table I.

### PH, alkalinity and turbidity values

**Figure 2:**
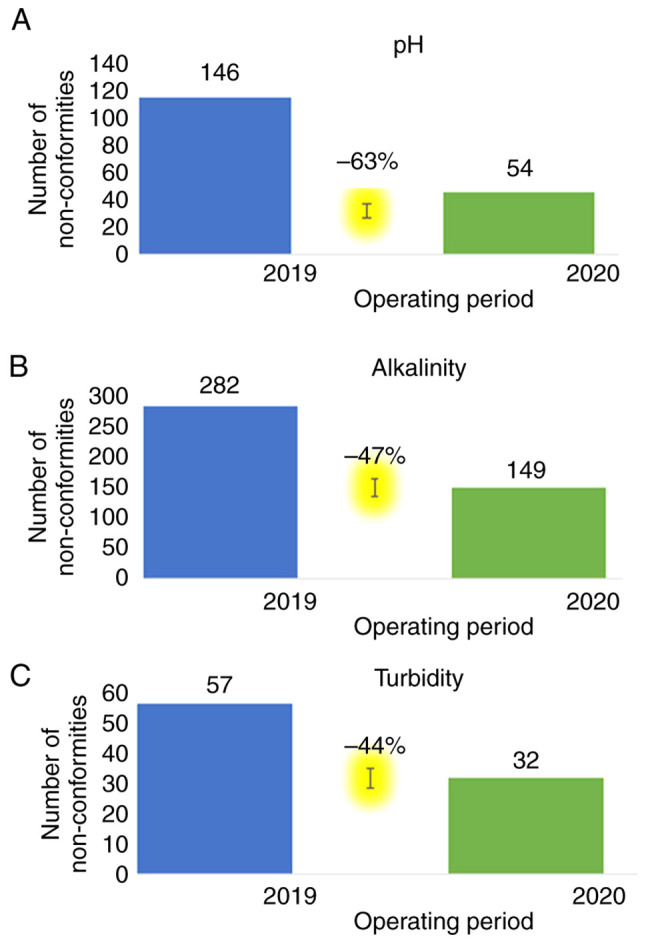
Reduction in non-conformities comparing the years 2019 and 2020 as regards (A) pH, (B) alkalinity, and (C) turbidity.

For the year 2019, the pH was measured in 381 samples. Of these, 235 (61.68%) were within acceptable limits (<7.2 and >7.8), while 146 (38.32%) were not. In 2020, out of the 210 samples measured, 156 (74.29%) were within limits, while 54 (25.71%) were not. A comparison of the pH values over the two time periods revealed a 63% reduction in non-conformities in the year with the strictest protocols (Fig. 2A). As regards the effect of the inappropriate pH on effective chlorination, the RR was found to be relatively high (RR=7.22; P=0.02) in 2019; however, due to both the relatively smaller number of samples and the high chlorination, it was found that there was no RR (RR=0.67; P=0.26) as regards the effect of the inappropriate pH on effective chlorination in 2020 (Table II).

In relation to the alkalinity of the samples for the year 2019, 99 (25.98%) samples were within, while 282 (74.02%) were out of the acceptable limits. For 2020, 61 (29.05%) samples were within limits, while 149 (70.95%) were not. It was found that there was a 47% decrease in non-conformities in 2020 when strict protocols were applied (Fig. 2B).

As regards turbidity, in 2019, 323 (84.78%) samples were found within limits, while only 58 (15.22%) were not. In 2020, 177 (84.29%) were within the acceptable limits, while 33 (15.71%) were not. It was observed that there was a 44% decrease in non-conformities in 2020 when strict protocols were applied (Fig. 2C).

### Microbiological parameters of water samples

The following limits were taken into account for the comparison of water microbiological parameters: *E. coli* and *P. aeruginosa, <*1 CFU/100 ml; *Legionella spp.* ≥50 CFU/l; total microbial flora, *<*200 CFU/ml; and total coliforms, >15 CFU/100 ml.

### Legionella spp

Of the 132 samples from 14 hotels collected in total, 49 samples were obtained in 2019 and 83 were obtained in 2020. In the 2019 tourist season, 47 (95.92%) samples were negative, while 2 (4.08%) were positive (*≥*50 CFU/l) for the presence of *Legionella spp.* In 2020, 76 (91.57%) samples were found to be negative, while 7 (8.43%) were positive (*≥*50 CFU/l). The small increase in the period of 2020 can be explained by the fact that the hotels remained closed for a long period of time, and thus the water circulating in the internal networks was stagnant, there was no continuous chlorination during the time they were closed, nor was the required attention given in relation to the *Legionella spp*., since all preventive measures were focused on the SARS-CoV-2 virus.

### E. coli

Of the 591 samples obtained in total over a period of 2 years, 577 (97.63%) were within limits, and 14 (2.37%) were not. In 2019, out of 381 samples, 370 (97.11%) were found within limits and 11 (2.89%) were not.

**Figure 3:**
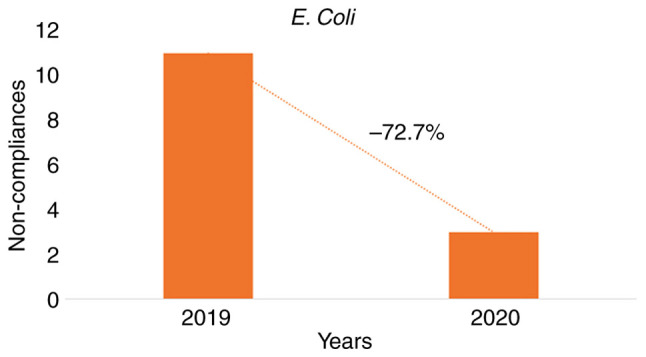
Reduction in non-conformities comparing the years 2019 and 2020 in relation to the presence of the pathogen *E. coli* in the water samples.

In 2020, of the 210 samples obtained, 207 (98.57%) were found within acceptable limits and 3 (1.43%) were not. Non-compliance with legislative requirements decreased by 72.7% between the two periods (Fig. 3).

As regards the impact of non-compliance with residual chlorine values on the increased presence of *E. coli* in the water of the swimming pools for the year 2019, the RR was calculated at 8.50 (P=0.0002), while for the year 2020, the RR was calculated at 14.50 (P=0.0215) (Table II).

Furthermore, in both reference years, the linear correlation coefficients revealed there was a weak correlation between the ineffective chlorination (<0.4 mg/l) and the unacceptable presence of *E. coli* (2019: r=0. 04, P=0.0001; 2020: r=0.00, P=0.7765) (Table III).

**Figure 4:**
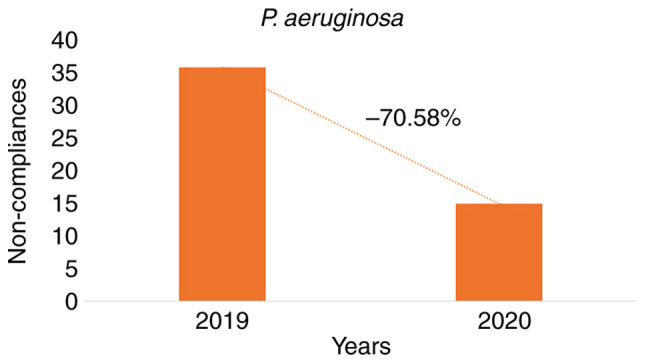
Reduction in non-conformities comparing the years 2019 and 2020 in relation to the presence of the pathogen *P. aeruginosa* in the water samples.

*P. aeruginosa.* A total of 591 samples were tested, of which 540 (91.37%) were found within acceptable limits and 51 (8.63%) were not. In 2019, 381 samples were tested, of which 345 (90,55%) were within acceptable limits and 36 (9,45%) were not. In 2020, 210 samples were tested, of which 195 (92.86%) were within acceptable limits and 15 (7.14%) were not. When the results of the two periods were compared, the legislative deviation was reduced by 70.58% (Fig. 4). The RR regarding the presence of *P. aeruginosa* due to the incorrect compliance with the requirements for residual chlorine in 2019 was 2.04 (P=0.0814), while in 2020 it was 2.07 (P=0.45) (Table II). Furthermore, in both reference years, the linear correlation coefficients revealed a weak correlation between the ineffective chlorination (<0.4 mg/l) and the unacceptable presence of *P. aeruginosa* (2019: r=0.04, P=0,0001; 2020: r=0.00, P=0,7210) (Table IV).

### Total microbial flora and total coliforms

In 2019, 381 samples were tested, of which 45 (11.81%) exhibited a deviation from the requirements of the relevant legislation for total microbial flora (>200 CFU/ml) and 12 (3.15%) for total coliforms (>5 CFU/100 ml) (Table III). These results revealed a decrease in non-conformities for both indicators in 2020 (82 and 75%, respectively). Furthermore, the lack of satisfactory chlorination in swimming pool water was associated with a high RR for the unacceptable presence of total microbial flora and total coliforms in both the years 2019 and 2020 (Table V).

## Discussion

With the onset of the COVID-19 pandemic, numerous countries issued stricter regulations and implemented additional measures to limit the spread of COVID-19 through activities in recreational water areas (5,17) .

The reopening of the hotels and the use of swimming pools under strict protocols were based on legislation and guidelines from the competent Ministries of Health and Tourism of Greece that did not substantially differ from the regulations of other countries or the instructions of the WHO (4). The stricter safety regulations during the use of swimming pools, greater vigilance, employee training, self-inspection by owners, and more systematic inspections by public health authorities have been shown to result in greater safety for users (22).

In the design, construction and operation of swimming pools, preventive measures should be taken into account to avoid the spread of diseases and ensure the comfort of users. During the operation of the pool, disinfection and filtration of the pool water are necessary for the destruction of microorganisms and the removal of contaminants, respectively (22). In numerous swimming pools, chlorine- or bromine-based disinfectants are used to prevent the growth of microbes. However, chlorination is the most widely used method (23). Chlorination is critical in order to ensure that the pools are free of microorganisms and safe for swimmers. Therefore, testing for free chlorine and maintaining proper chlorine levels in swimming pools is an important safety parameter.

In this study, chlorine was used exclusively by the operators of the swimming pools studied as the main disinfectant method. Studies have demonstrated that water samples with incompatible free chlorine levels and a pH >7.8 were outside legislative limits regarding bacteria, as reflected in the present study (24-26). The main cause of recreational water contamination and, therefore, the greatest risk to public health associated with bathing derives from microorganisms in contaminated feces.

The present study demonstrated that the increased levels of residual chlorine measured due to the requirements of health protocols had a significant effect on the microbiological hygiene of the water in the swimming pools studied. This is confirmed by the high compliance rate in all parameters, apart from *Legionella spp.* Its increased presence in internal water distribution networks is scientifically proven as chlorination does not, on its own, have a great impact on its growth. Non-conformity rates decreased in the year 2020 for the parameter total microbial flora at 36˚C x 24 h at a rate of 82%, for total coliforms at a rate of 75%, for the presence of *E. coli* at a rate of 72.7%, and at a rate of 70.58% for the presence of *P. aeruginosa*.

The quality of the pool water is dependent on several factors, such as the degree of water pollution, the number of bathers and their behavior, the ventilation system (in the case of closed tanks), the temperature of the water and air and the water circulation system. The results of physicochemical tests of pool water quality parameters, carried out by health inspection bodies, are considered auxiliary to the assessment of its degree of contamination (27). The results of microbiological examinations and the assessment of the sanitary condition of the facility play a decisive role. Even prior to the start of the pandemic, strict standards were in place for water treatment and maintaining its quality in swimming pools (25). Water should be filtered and disinfected and should meet the physical, chemical, and microbiological properties defined by Greek legislation. This current legislation also determines the required monitoring frequencies for physicochemical and microbiological indicators and the procedures for closing and maintaining the tank in case of non-compliance (26).

Disinfectants added to water should kill or inactivate microorganisms, while maintaining the integrity of the skin, eyes and mucous membranes of swimmers. Chlorine is commonly used due to its cost-effectiveness, easy use and safety. Cleaning and disinfection measures should be accompanied by strict rules of hygiene and behavior for bathers, as well as a limit on the number of bathers allowed (10).

It has been demonstrated that as far as bacterial pathogens are concerned, both total coliforms and *E. coli* are detected in ~10% of the swimming pools studied, presenting similar percentages to the present study for the year 2019, but were reduced in the year 2020 due to strict protocols that required high chlorine levels and a reduced number of swimmers. These indicator organisms can potentially cause various diseases, particularly in children <5 years of age and the elderly (28-30).

A 20-year review of the epidemiology of *E. coli O157:H7* in the USA revealed a total of 350 outbreaks, with 31 (9%) related to recreational water, a third of which are connected to swimming pools. The presence of these organisms in the water often indicates the inefficiency of water disinfection, particularly in pools with high total concentrations of coliforms (30,31).

The present study found low rates of the pathogenic bacterium, *P. aeruginosa*, in both the reference years 2019 and 2020, in contrast to other studies, in which it was found to be particularly widespread (69%) (17,32,33). *P. aeruginosa* is present in water and is resistant to chemical disinfectants, such as chlorine. *P. aeruginosa* causes a variety of diseases, including bursitis, otitis externa, keratitis, urinary tract infections and gastrointestinal infections through exposure to the skin, ears, eyes, urinary tract, lungs and intestine. In total, 79% of ear infections in swimmers are attributed to *P. aeruginosa*, with symptoms ranging from ear pain to hearing loss (32,33). According to a survey of water-borne diseases in the USA between 1999 and 2008, *P. aeruginosa* was the second most common water-borne pathogen following *Cryptosporidium* (34).

The pH of pool water should be examined to ensure effective disinfection and user comfort. The pH should be maintained between 7.2 and 7.8 for chlorine disinfectants (16). Public health bodies worldwide report that, if the pH is <7.2, there is the possibility of discomfort in the eyes due to accelerated chloramine formation, rapid chlorine loss, the etching of exposed cement and the corrosion of metals. If the pH is >7.6, there is a possibility of reducing the effectiveness of disinfection with chlorine, increasing the chlorine requirement, discomfort in the eyes, drying of the skin, turbid water, and scale formation (35). The study by Masoud *et al* (24) observed that the majority of swimming pools did not follow the accepted pH standard. In the present study, 38.32% of samples in 2019 and 25.71% of samples in 2020 were out of the acceptable limits.

As regards chlorine as a disinfectant in the water of swimming pools, according to the study by Mellou *et al* (5), which used a questionnaire to investigate the observance of strict health protocols in hotel swimming pools during the COVID-19 pandemic, the level of free chlorine in the pool water defined by the regulations (1-3 mg/l) was achieved in 70% of the facilities, a percentage that matches the present study, where a 75% compliance rate at a minimum level of residual chlorine (1.5 mg/l) was observed.

As regards the findings of the present study in relation to the increase in the positivity of water samples from swimming pools for *Legionella spp.*, the prevention guidelines state that the water in the internal network of hotels should not be standing, it is required to be sufficiently chlorinated and a strict water safety plan should be applied (36,37). The presence of the bacterium *Legionella spp.* in hotel water distribution networks, as well as in recreational waters is one of the most severe microbiological hazards that only a comprehensive water safety plan can effectively address.

In conclusion, the stricter safety measures during the COVID-19 pandemic for the safe operation of swimming pools led to a significant improvement in terms of legislative compliance on physicochemical parameters, as well as microbiological indicators in the recreational waters of the hotels studied. Greater vigilance, self-control and incentives for swimming pool managers and operators, as well as more systematic inspections and sampling by competent public health authorities, can lead to safer pool operation.

The modernization of swimming pool operating regulations, both in terms of risk analysis and the addition of legislative requirements for limit compliance and other mandatory microbiological indicators, such as *P. aeruginosa, Cryptosporidium* and the use of *Staphylococcus aureus* as a supplementary indicator when appropriate are of utmost importance. In addition, according to international experience, it is required to change the permissible pH limit in the current Greek legislation from 7.2-8.2 to 7.2-7.8 for more effective disinfection and a reduction of the side-effects of chlorination.

Finally, a strong recommendation is required in order to implement water safety plans in hotel units, given that, already in 2004, the WHO guidelines recommended that water suppliers develop and implement ‘water safety plans’ in order to systematically assess and manage risks. This arrangement will contribute significantly to the immediate and effective management of emerging public health issues and issues regarding recreational waters (*Legionella spp.*, *P. aeruginosa*, *Cryptosporidium, Staphylococcus aureus*, etc. pathogens).

## Figures and Tables

**Table I:** Comparison of residual chlorine values <0.4, <0.7, <1 and <1.5 mg/l between 2019 and 2020, and the estimated reduction in non-conformities.

	2019		2020		
Variable	No. of samples	%	No. of samples	%	% Reduction in non-conformities
Residual chlorine <0.4 mg/l	34	8.92	7	3.33	79
Residual chlorine <0.7 mg/l	60	15.75	16	7.62	73
Residual chlorine <1 mg/l	101	26.51	25	11.90	75
Residual chlorine <1.5 mg/l	175	45.93	52	24.76	70

**Table II:** Calculation of relative risks regarding studied parameters in 2019 and 2020.

	2019			2020		
Parameter	Relative risk	P-value	95% CI	Relative risk	P-value	95% CI
Risk of exceeding limits regarding the presence of *E. coli* due to ineffective chlorination (<0.4 mg/l)	8.50	0.000	13.841 (Harm) to 5.339 (Harm)	14.50	0.02	22.016 (Harm) to 4.533 (Harm)
Risk of exceeding limits regarding the presence of *P. aeruginosa* due to ineffective chlorination (<0.4 mg/l)	2.04	0.0814	5.191 (Harm) to to 79.275 (Benefit)	2.07	0.45	3.736 (Harm) to ∞ to 8.340 (Benefit)
Risk of exceeding limits regarding the presence of *total microbial flora* due to ineffective chlorination (<0.4 mg/l)	3.30	0.0001	8.760 (Harm) to 2.968 (Harm)	4.14	0.15	3.970 (Harm) to 28.479 (Benefit)
Risk of exceeding limits regarding the presence of *total coliforms* due to ineffective chlorination (<0.4 mg/l)	7.28	0.0004	14.992 (Harm) to 5.346 (Harm)	3.64	0.37	7.079 (Harm) to 19.772 (Benefit)
Risk of ineffective chlorination (<0.4 mg/l) due to inappropriate pH	0,67	0.26	39.760 (Harm) to 10.820 (Benefit)	2.535	0.26	18.993 (Harm) to 74.865 (Benefit)

**Table III:** Results of correlation analysis of *E. coli* and inadequate chlorination in 2019 and 2020.

2019 Coefficient: r^2^=0.04					
Source	df	Sum of squares	Mean square	F-statistic	P-value
Regression	1	1154.2009	1154.2009	15.3880	0.0001
Residuals	379	28427.4107	75.0064		
Total	380	29581.6115			
Pearson's correlation analysis			Spearman's correlation analysis		
Coefficient	T statistic	P-value	Coefficient	T statistic	P-value
0.1975	3.9228	0.0001	0.2218	4.4279	<0.0001
2020 Correlation coefficient: r^2^=0.00					
Source	df	Sum of squares	Mean square	F-statistic	P-value
Regression	1	3.8438	3.8438	0.0808	0.7765
Residuals	208	9893.9704	47.5672		
Total	209	9897.8143			
Pearson's correlation analysis			Spearman's correlation analysis		
Coefficient	T statistic	P-value	Coefficient	T Statistic	P-value
0.0197	0.2843	0.7765	0.2001	2.9459	0.0036

**Table IV:** Linear correlation analysis of *P. Aeruginosa* and inadequate chlorination in 2019 and 2020.

2019 Coefficient: r^2^=0.04					
Source	df	Sum of squares	Mean square	F-statistic	P-value
Regression	1	206.9233	206.9233	14.7684	0.0001
Residuals	379	5310.2421	14.0112		
Total	380	5517.1654			
Pearson's correlation analysis			Spearman's correlation analysis		
Coefficient	T statistic	P-value	Coefficient	T statistic	P-value
0.1937	3.8430	0.0001	0.0919	1.7964	0.0732
2020 Coefficient: r^2^=0.00					
Source	df	Sum of squares	Mean square	F-statistic	P-value
Regression	1	10.5938	10.5938	0.1279	0.7210
Residuals	208	17233.9015	82.8553		
Total	209	17244.4952			
Pearson's correlation analysis			Spearman's correlation analysis		
Coefficient	T statistic	P-value	Coefficient	T statistic	P-value
-0.0248	0.3576	0.7210	0.0461	0.6651	0.5067

**Table V:** Frequency of total microbial flora and total coliforms within and out of acceptable limits in 2019 and 2020.

Total microbial flora	No.	%
2019		
Within limits	336	88.19
Out of limits	45	11.81
2020		
Within limits	202	96.19
Out of limits	8	3.81
Total coliforms	No.	%
2019		
Within limits	369	96.85
Out of limits	12	3.15
2020		
Within limits	207	98.57
Out of limits	3	1.43

## Data Availability

The datasets used and/or analyzed during the current study are available from the corresponding author on reasonable request.
